# A route to possible civil engineering materials: the case of high-pressure phases of lime

**DOI:** 10.1038/srep12330

**Published:** 2015-07-23

**Authors:** A. Bouibes, A. Zaoui

**Affiliations:** 1 LGCgE, Polytech’Lile, University of Lille1. Cité Scientifique, Avenue Paul Langevin, 59655 Villeneuve d’Ascq, France

## Abstract

Lime system has a chemical composition CaO, which is known as thermodynamically stable. The purpose here is to explore further possible phases under pressure, by means of variable-composition *ab initio* evolutionary algorithm. The present investigation shows surprisingly new stable compounds of lime. At ambient pressure we predict, in addition to CaO, CaO_2_ as new thermodynamically stable compound. The latter goes through two phases transition from C2/c space group structure to Pna21 at 1.5 GPa, and Pna21 space group structure to I4/mcm at 23.4 GPa. Under increasing pressure, further compounds such as CaO_3_ become the most stable and stabilize in P-42_1_m space group structure above 65 GPa. For the necessary knowledge of the new predicted compounds, we have computed their mechanical and electronic properties in order to show and to explain the main reasons leading to the structural changes.

Lime is one of the most important and largely used building materials. It is used in several ways in civil engineering. It can be used alone, hydrated and also by mixing it with other civil engineering materials to perform their properties. Lime is basically known thermodynamically stable as a calcium oxide (CaO). It is usually made by the decomposition of materials that contain calcium carbonate by decomposition reaction: CaCO_3_ = CaO + CO_2_. The stability and structural properties of the mineral parent (calcite) has been subject of intense investigations^1^,^2^,^3^,^4^. However, less is known about further possible phases of lime.

The goal of the present work is to investigate new thermodynamically stable compounds from lime system. New stable stoichiometries could have, indeed, important planetological and chemical implications.

Calcium oxide, CaO, is one of the most known thermodynamically stable compounds of Ca-O system at ambient conditions. This is one of the most abundant compounds in the planetary mantles after MgO, SiO_2_ and FeO, which are considered as the building blocks of the mantle minerals^5^. Several experimental and theoretical investigations of high-pressure structure and phase stability of CaO have been done, resulting that, CaO crystallizes in the NaCl-type structure (B1) and transforms into CsCl-type structure (B2) around 62 GPa. The corresponding ground state properties have been widely investigated both experimentally and theoretically^5^,^6^,^7^.

On the other hand, a recent theoretical study shown that calcium peroxide, CaO_2_, is a thermodynamically stable composition in Pna2_1_ space group structure at ambient conditions^8^. Mumtaz *et al.* performed experimentally the structural properties of calcium peroxides. They observed a tetragonal structure for CaO_2_^9^. However, apart of this, much is still to be known about its fundamental properties as well as its behavior under pressure effect.

In the present study, we aim to investigate the new thermodynamically stable compounds based on Ca-O composition. To this end, we will explore all stable compositions from Ca-O system and their crystal structures at high pressures using the variable and fixe composition *ab initio* evolutionary algorithm^10^. Besides, we will discuss their different structures, phase transitions, elastic properties and chemical bonding.

In order to find all possible phases of lime and their corresponding structures, we used the *ab initio* evolutionary algorithm (USPEX)^10^,^11^,^12^, which has a capability to simultaneously find stable stoichiometries and the corresponding structures in multicomponent systems. In our calculations, we allowed all possible compositions in our studied system with structures containing up to 10 atoms in the unit cell. The calculations were performed at zero Kelvin and pressures of 1 atm, 50 GPa, 70 GPa and 100 GPa. The initial generation consisted of 120 structures, and all subsequent generations have 40 structures. Stable structures and their compositions were determined using the convex hull construction. A compound is thermodynamically stable if the enthalpy of its decomposition into any other compound is positive^10^.

The present investigation results a surprisingly new stable compounds of lime. While we reproduce stability of CaO in the whole pressure range investigated here, new stable compounds are predicted. In Fig. 1, we display the convex hull of Ca-O system at high pressure. The enthalpies of formation of the predicted structures are shown as well. It can be clearly seen that in addition to CaO, CaO_2_ is thermodynamically stable at high pressure.

At 70 GPa, convex hull diagram shows that a new compound, namely CaO_3_, becomes thermodynamically stable. The latter remains thermodynamically stable up to 100 GPa (Fig. 1). The latter have never been reported before.

Thereafter, we explore all our results of stable compounds for lime, using fixe composition evolution algorithm, in order to determine the possible phase change under pressure. The structure prediction runs for CaO and CaO_2_ compound were performed at 1 atm, 20 GPa, 50 GPa, 70 GPa and 100 GPa, for CaO_3_ at 70 GPa, all at zero Kelvin. The phase diagram and crystal structures of the newly predicted compounds are remarkable. The pressure-composition phase diagram is shown in Fig. 2. We may see that CaO phase transition occurs at around 65 GPa. It is stable below 65 GPa in Fm-3 m space group structure. Above 65 GPa, the structure of Pm3m space group becomes more stable. This is in good agreement with both experimental and theoretical data^5^,^6^,^7^. On the other hand, calcium peroxide, CaO_2_, stabilize in C2/c space group structure (Fig. 3a) at ambient conditions. At 1.5 GPa, CaO_2_ has a first phase transition. Pna2_1_ space group structure (Fig. 3b), reported by Zhao *et al.*^8^, becomes the most stable between 1.5 GPa and 23.4 GPa. The second phase transition of CaO_2_ occurs at 23.4 GPa. Above 23.4 GPa, a new I4/mcm structure (Fig. 3c) is predicted to be stable. CaO_3_ stabilizes in P-42_1_m space group structure above 65 GPa (Fig. 4). Each Ca atom is bonded with four series of O_3_ units, and each oxygen atom is bonded with four atoms of calcium. The structural properties for all these compounds are reported in Table 1.

In order to confirm the stability of the obtained structures at different pressures, we list in Table 2 the enthalpies per formula unit of each compound with the one of oxygen. For instance, the enthalpy of the decomposition reaction ∆E of CaO_2_, from a reaction CaO + 1/2 O_2_ −>CaO_2_, is defined as:


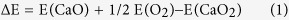


E (CaO), E (CaO_2_) and E (O_2_) are enthalpy per formula unit of the oxide CaO, CaO_2_ and isolated oxygen molecule. Positive reaction enthalpy indicates a thermodynamically stable compound. Furthermore, a compound is thermodynamically stable if the enthalpy of its decomposition into any other compounds is positive. A compound is thermodynamically stable if the enthalpy of its decomposition into any other compounds is positive. Indeed at 1atm, we obtain E (CaO) = −12.95 eV per f.u. and E (O_2_) = −9.94 eV per molecule. The decomposition energy ∆E of the CaO_3_ is −0.69 eV, indicating that it is not stable at ambient pressure.

Mechanically, the elastic constants of a material describe its response to an applied stress or, conversely, the stress required to maintain a given deformation. These properties are obviously directly related to the mechanical stability of a given system. The criteria of the mechanical stability of a monoclinic crystal are as follows^13^:


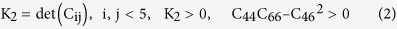


and of tetragonal crystal are^13^:


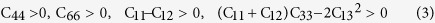


The calculated elastic constants of CaO_2_, CaO_3_, at 1atm, 65 GPa respectively are listed in Table 3. The above mentioned criteria are satisfied in our case, indicating that CaO_2_, CaO_3_ are mechanically stable at the corresponding conditions. For comparison, the values of the elastic properties of CaO are presented as well, and we notice the excellent agreement with the reported data. We may also underline that calcium oxide is more rigid and more resistant to shear deformation than calcium peroxide at the ambient conditions. Moreover, the Pugh modulus ratio^14^ *k* = G/B is also reported in Table 3 to show the ductile-brittle behavior of high pressure lime phases. For brittle materials, G/B is higher than 0.571, whereas, for the ductile ones, it is lower than 0.571. It follows that CaO and CaO_3_ are brittle; while CaO_2_ is ductile. According to the following equation of hardness^15^:


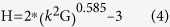


The Vickers hardness H of CaO, CaO_2_, and CaO_3_ are 13.75, 4.28 and 16.88 GPa respectively, suggesting that the harder phase is CaO_3_ and the less hard is CaO_2_.

In order to better understand the bonding behaviors, a Bader charge analysis^16^ of the calculated charge densities is carried out. At ambient pressure, Bader charge analysis shows that each Ca atom gives 1.47 electrons to each O atom in CaO. In the case of CaO_2_, Ca atom gives 1.56 electron/atom and O atom gets −0.78 electron/atom. At 65GPa, the charge configuration of CaO_3_ is Ca^+1.47^ [O^−0.62^O^−0.23^O^−0.62^], the partial electronic charge transfer is not symmetric from the Ca to O atoms. We can also notice that the electronic charge of Ca atom is approximately the same for all phases. Besides, we have performed the total density of state for all stable phases of lime, as represented in Fig. 5. For comparison, we calculate the band gap value of CaO (3.65 eV), which is in perfect agreement with previous work^7^. The obtained band gap value of CaO_2_, CaO_3_ is 2.78 eV and 2.48 eV respectively. Additionally, magnetic calculation is performed for CaO_3_ compound. The calculation shows that there is no magnetic moment on all atoms of this compound, what means that CaO_3_ is non-magnetic.

In the studied pressure range, calcium peroxide knows two phase transitions at 1.5 GPa and at 23.4 GPa. The most stable CaO_2_ at ambient conditions corresponds to the space group C2/c. Zhao *et al.* found that CaO_2_ stabilizes in Pna2_1_ space group structure^8^. However, the last structure becomes stable above 1.5 GPa of pressure. After duplicating both structures, we may notice that those structures are very similar as shown in Fig. 2. In Table 4, we compare the atomic distances between the obtained structure and the one of Zhao. We can see that the atomic distances are very close.

Calcium peroxide goes through a second phase transition from Pna2_1_ space group structure (Fig. 3b) to I4/mcm space group structure (Fig. 3c) at 23.4 GPa. The tetragonal structure with unit cell parameters a = 5.01 Å and c = 5.92 Å is observed experimentally at a temperature of 550 °C (823 K) by Mumtaz *et al.*^9^. The corresponding lattice parameters are very similar with our findings. Additionally, we notice that the discovered MgO_2_ stabilizes also in the same structure with I4/mcm space group structure above 500 GPa^17^.

In order to understand the main reasons behind CaO_2_ phase transition, let us analyze in the bonding character through the electronic charge density. Figure 6 represents the electronic charge density of CaO_2_ compound at 1 atm and 20 GPa in the (1 1 1) plane, which contain the maximum Ca and O atoms. We can remark that the electronic structure change between 1atm and 20 GPa. Consequently, new bond between calcium and oxygen are formed at 20GPa leading to a new phase. This explains and justifies the main reasons behind the transitions.

In summary, we have explored new phases of lime through variable-composition *ab initio* evolutionary algorithm. At ambient pressure we predicted, in addition to CaO, CaO_2_ as new thermodynamically stable compound under two transitions at 1.5 GPa and at 23.4 GPa. Further compounds have been predicted such as CaO_3_ above 65 GPa.

For the more complete knowledge on the new predicted compounds, we have investigated various kinds of properties including mechanical, electronic and bonding to explain the main reasons leading to the transitions giving the new class of materials from the simple lime system.

## Methods summary

We use here the “Universal Structure Predictor: Evolutionary Xtallography” (USPEX) code^10^,^11^,^12^. The latter is based on approach features global optimization with real-space representation and physically motivated variation operators. To generate every candidate structure, we use first-principles structural relaxation, based on density functional theory within the GGA functional^17^ for solids^18^,^19^.

We used the projector-augmented wave (PAW)^20^ with Ca [3s^2^3p^6^4s^2^] and O [2s^2^2p^4^] cores (core radii 2.00 a.u. and 1.5 a.u., respectively). The plane-wave kinetic-energy cutoff is 600 eV, and the k-point mesh resolution in reciprocal space is 2 * 0.06 A^−1^. These settings enable excellent convergences of the energy differences, stress tensors, and structural parameters. The predicted structure calculation was performed with fixed composition. The plane-wave kinetic-energy cutoff is also 600 eV, and the k-point mesh resolution in reciprocal space is 2 * 0.03 A^−1^.

## Additional Information

**How to cite this article**: Bouibes, A. and Zaoui, A. A route to possible civil engineering materials: the case of high-pressure phases of lime. *Sci. Rep.* **5**, 12330; doi: 10.1038/srep12330 (2015).

## Figures and Tables

**Figure 1:**
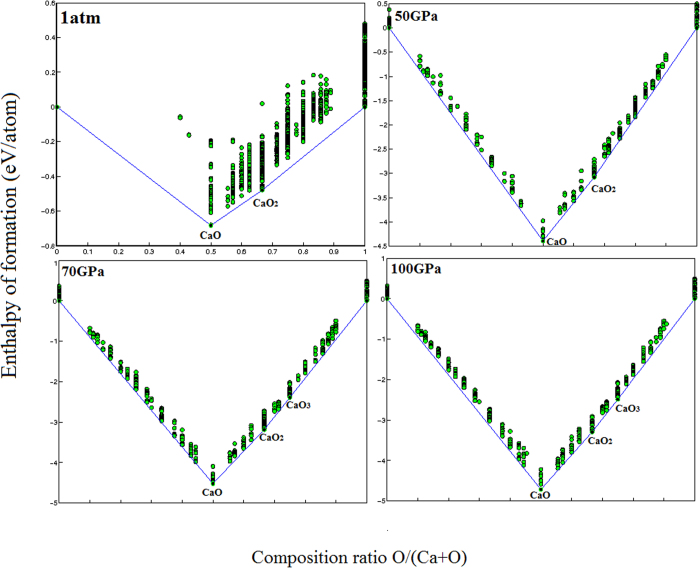
Convex hull diagrams for the Ca – O system at different pressure. (**a**) 1atm; (**b**) 50 GPa, (**c**) 70 GPa and (**d**) 100 GPa.

**Figure 2:**
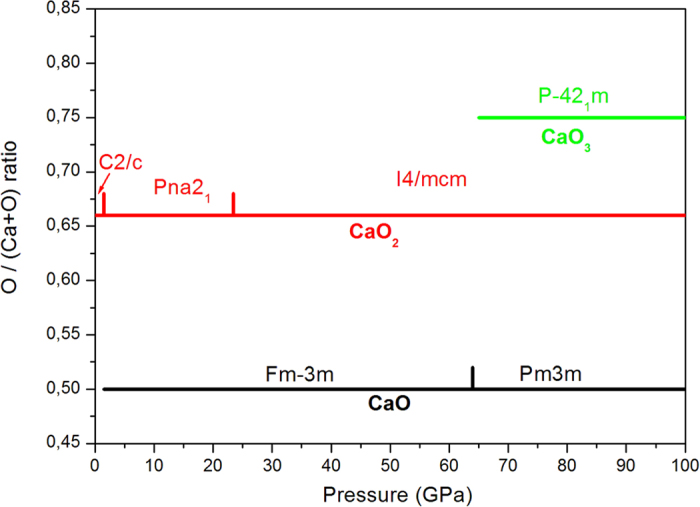
Pressure composition phase diagram of Ca-O system.

**Figure 3:**
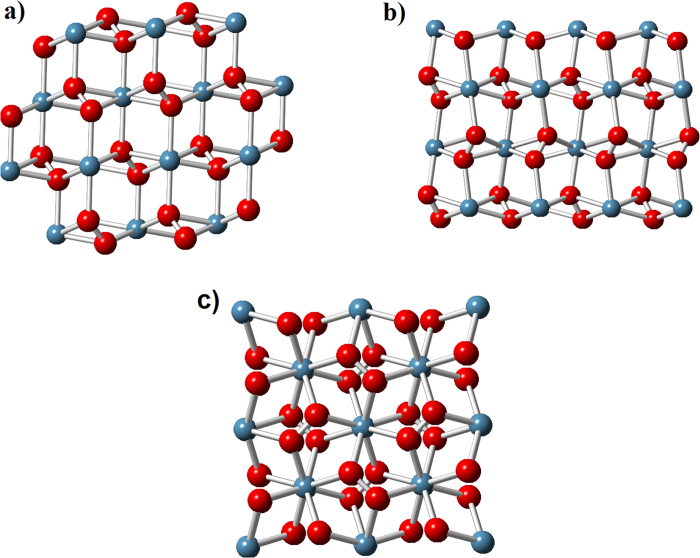
CaO_2_ structures. (**a**) CaO_2_- C2/c (**b**) CaO2-Pna2_1_ (**c**) CaO_2_ – I4/mcm. Large blue spheres –Ca atoms, small red spheres – O atoms.

**Figure 4:**
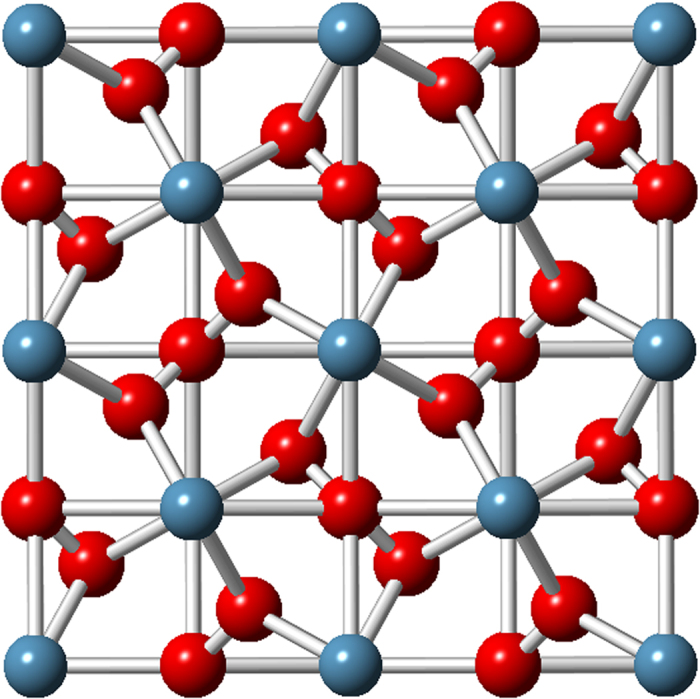
Crystal structure CaO_3_. Large blue spheres –Ca atoms, small red spheres – O atoms.

**Figure 5:**
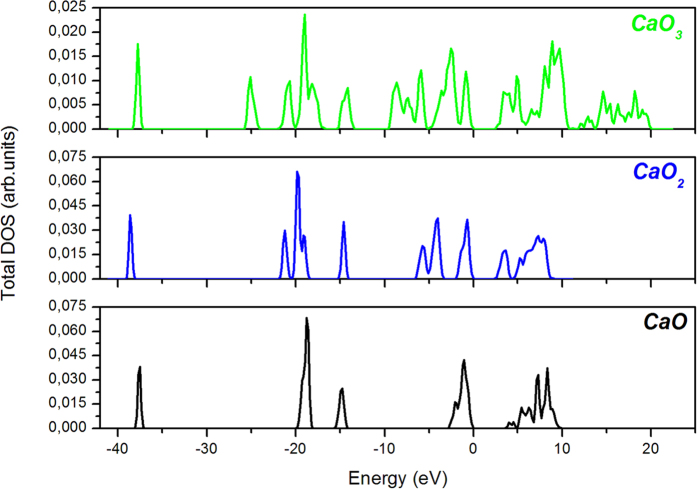
Total DOS for each phase of Ca-O for various compositions. The Fermi level is set to zero.

**Figure 6:**
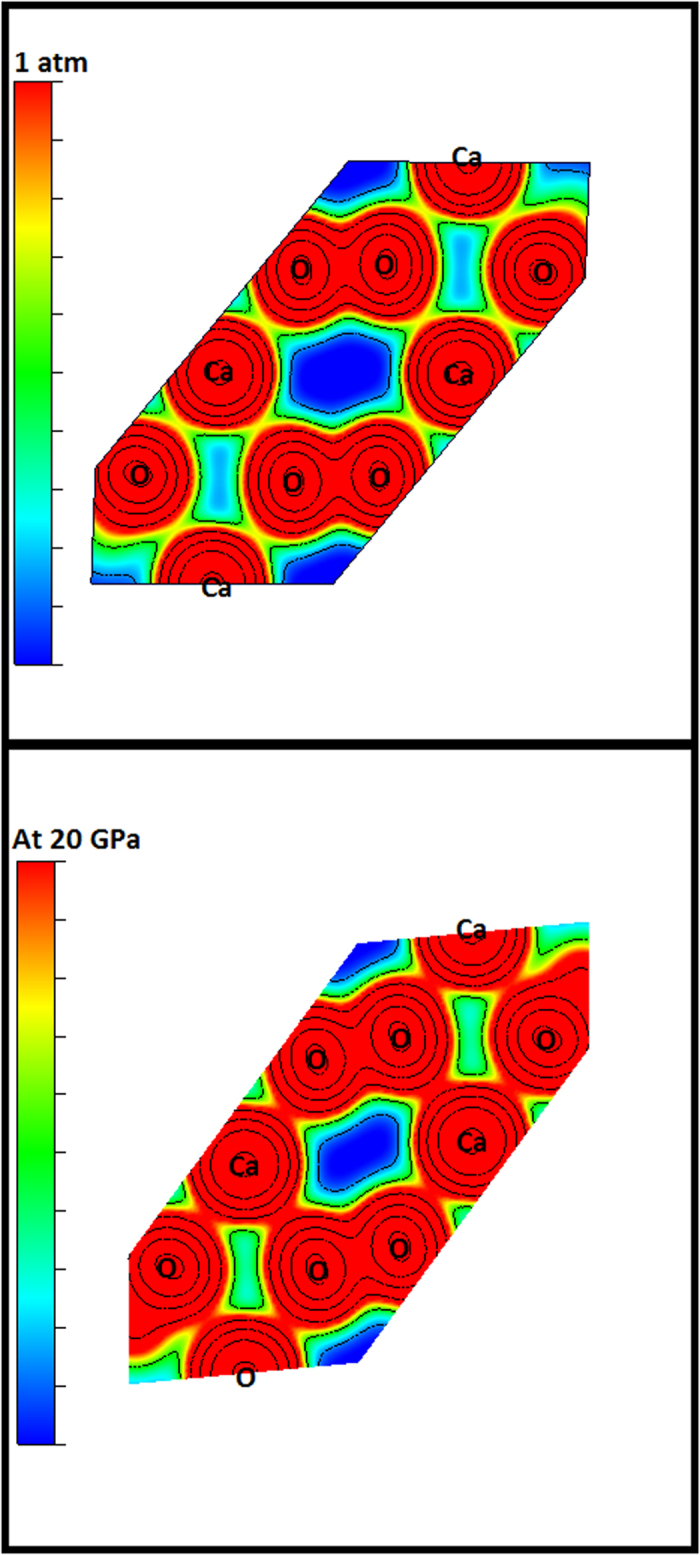
Valence charge density of CaO_2_-Phase1 along the (1 1 1) plane at 1atm and 20 GPa.

**Table 1:** Structural properties and entropy of stable phases of lime.

Structure	Space group	Lattice Parameters (Å)		X	Y	Z	
*CaO-Phase1*	Fm-3m	a = 4.8	Ca	0.00	0.00		0.00
			O	0.50	0.00		0.00
*CaO-Phase2*	Pm3m	a = 2.56	Ca	0.00	0.00		0.00
			O	0.50	0.50		0.50
*CaO*_*2*_*-Phase1*	C2/c	a = 6.82	Ca	0.5	0.14		0.75
		b = 3.68	O	0.75	0.64		0.91
		c = 7.04					
		β = 117.9°					
*CaO*_*2*_*-Phase3*	I4/mcm	a = 4.78	Ca	0.00	0.00		0.75
		c = 6.15	O	0.11	0.39		0.50
*CaO*_*3*_	P-42_1_m	a = 4.68	Ca	0.00	0.00		0.00
		c = 2.84	O1	0.00	0.50		0.79
			O2	0.82	0.32		0.51

**Table 2:** Enthalpy of formation (eV) of lime phases at various pressure.

	E(O_2_)	E(CaO)	E(CaO_2_)	E(CaO_3_)
*1atm*	−9.94	−12.95	−17.96	−22.20
*50GPa*	−4.52	−5.38	−8.09	−10.25
*70GPa*	−2.85	−2.83	−4.78	−6.46
*100GPa*	−0.51	0.48	−0.12	−0.81

**Table 3:** Mechanical properties of lime phases in GPa units.

	CaO		CaO_2_	CaO_3_
	*Fm-3m*	*Theory-Experiment*	*C2c*	*P-42*_*1*_*m*
C_11_	204.92	200^a^–221.89^b^	151.38	442.31
C_22_	206.51	—	136.95	442.90
C_33_	206.49	—	133.35	454.63
C_44_	74.68	66^a^–80.59^b^	38.83	226.16
C_55_	74.78	—	58.73	176.29
C_66_	74.78	—	53.49	174.29
C_12_	54.07	50^a^–57.81^b^	56.80	219.08
C_13_	53.91	—	69.89	177.54
C_23_	54.86	—	54.53	177.91
C_46_	−0.003		0.35	0.11
B	104.46	102^a^–110^b^	92.14	276.40
G	75.04	71^a^–80.59^b^	43.03	184.68
*k* = G/B	0.71		0.46	0.67
H	13.75		4.28	16.88

^a^Theory^14^,

^b^Experiment^13^

**Table 4:** Atomic distance at ambient conditions for C2/c – CaO_2_ structure and Pna2_1_- CaO_2_ structure.

	C2/c – CaO_2_	Pna2_1_ – CaO_2_
Ca – O	2.41; 2.42	2.38; 2.44; 2.42; 2.43; 2.47
O – O	1.51	1.51
Ca – Ca	3.66; 3.67	3.62; 3.64
